# Can adolescents with eating disorders be treated in primary care? A retrospective clinical cohort study

**DOI:** 10.1186/s40337-021-00413-9

**Published:** 2021-04-23

**Authors:** Jocelyn Lebow, Angela Mattke, Cassandra Narr, Paige Partain, Renee Breland, Janna R. Gewirtz O’Brien, Jennifer Geske, Marcie Billings, Matthew M. Clark, Robert M. Jacobson, Sean Phelan, Cynthia Harbeck-Weber, Daniel Le Grange, Leslie Sim

**Affiliations:** 1 Department of Psychiatry and Psychology, Mayo Clinic School of Medicine, 200 First Street SW, Rochester, MN 55905 USA; 2 Mayo Clinic Robert D. and Patricia E. Kern Center for the Science of Health Care Delivery, Rochester, MN USA; 3 Department of Pediatric and Adolescent Medicine, Mayo Clinic School of Medicine, Rochester, MN USA; 4 Department of Pediatrics, University of Minnesota, Minneapolis, MN USA; 5 Department of Health Science Research, Mayo Clinic School of Medicine, Rochester, MN USA; 6 Department of Psychiatry, University of California, San Francisco, San Francisco, CA USA; 7 Department of Psychiatry & Behavioral Neuroscience, The University of Chicago, Chicago, IL USA

**Keywords:** Feeding and eating disorders, Anorexia nervosa, Primary health care, Adolescent, Pediatrics

## Abstract

**Background:**

Family-Based Treatment (FBT) is considered the first-line intervention for adolescent anorexia nervosa. However, access to this treatment is limited. Treatment programs for other pediatric mental health conditions have successfully overcome barriers to accessing evidence-based intervention by integrating mental health services into primary care. This study evaluated the proof-of-concept of a novel modification of FBT, Family-Based Treatment for Primary Care (FBT-PC) for adolescent restrictive eating disorders designed for delivery by primary care providers in their practices.

**Methods:**

This retrospective clinical cohort study evaluated 15 adolescents with restrictive eating disorders receiving FBT-PC and 15 adolescents receiving standard FBT. We examined improvement in BMI percentile, reduction in weight suppression, and clinical benchmarks of eating disorder recovery including weight restoration to > 95% of expected body weight (EBW) and resolution of DSM-5 criteria for eating disorders.

**Results:**

In both groups, effect sizes for increased BMI percentile exceeded Cohen’s convention for a large effect (FBT-PC: *d* = .94; standard FBT: *d* = 1.15) as did effect sizes for reduction in weight suppression (FBT-PC: *d* = 1.83; standard FBT: *d* = 1.21). At the end of treatment, 80% of the FBT-PC cohort and 87% in the standard FBT group achieved > 95%EBW and 67% in the FBT-PC group and 60% in the standard FBT group no longer met DSM-5 criteria for an eating disorder. There were no cohort differences in the number of treatment drop-outs or referrals to a more intensive level of eating disorder treatment.

**Conclusions:**

Findings suggest that primary care providers have potential to improve weight and clinical status in adolescents with restrictive eating disorders. Based on these results, more rigorous testing of the FBT-PC model is warranted.

## Plain English summary

Family-Based Treatment (FBT) is considered the first-choice intervention for adolescent anorexia nervosa. However, finding a qualified provider in this modality is difficult. Treatment programs for other pediatric mental health conditions have successfully overcome barriers to accessing quality treatment by integrating mental health services into primary care. This study evaluated the proof-of-concept of a novel modification of FBT, Family-Based Treatment for Primary Care (FBT-PC) for adolescent restrictive eating disorders designed for delivery by primary care providers in their practices. This study examined weight gain in 15 adolescents with restrictive eating disorders receiving FBT-PC and 15 adolescents receiving standard FBT. At the end of treatment, both groups showed large improvements in BMI percentile and large decreases in weight suppression. Each group had the same number of drop-outs and referrals to more intensive eating disorder treatment. Findings suggest that primary care providers have potential to improve weight and clinical status in adolescents with restrictive eating disorders. Based on these preliminary results, more rigorous testing of the FBT-PC model is indicated.

## Introduction

Over the last two decades, the field of eating disorders has made considerable progress in the development and evaluation of evidence-based treatment for adolescent eating disorders. Family-Based Treatment (FBT) for anorexia nervosa [1] has amassed robust evidence supporting its effectiveness [2–6]. Despite this support, few FBT providers exist, with only 85 certified providers globally [7]. As such, there are significant gaps in treatment access, especially for those in low-resourced areas. This likely contributes to substantial delays in care [8], which have been shown to lead to more severe illness and poorer outcomes [9–11].

The scarcity of trained providers is not unique to eating disorders. For adolescents across psychiatric diagnoses, there are similar difficulties accessing mental health services [12, 13]. By providing multidisciplinary behavioral healthcare embedded within the primary care setting, integrated behavioral health programs have successfully helped narrow these gaps [14, 15] and have shown benefit for adolescent depression [16], pediatric behavior problems [17], and attention-deficit/hyperactivity disorder [18].

Because eating disorders are complex conditions with both medical and psychological features that require clinical attention, they lend themselves well to an integrated care model. Moreover, since the majority of adolescents with eating disorders present first to primary care [19], embedding treatment at this point of care may improve rates of early detection and effective intervention [20]. In addition, patients often delay or avoid presenting for specialty eating disorder care due to the high degree of stigma and pervasive misunderstandings surrounding these disorders [21]. Basing eating disorder treatment in a primary care medical setting as opposed to a mental health center may improve patient retention and compliance by emphasizing the biological aspects of the condition while minimizing its association with mental health disorders, which have been historically stigmatized.

In light of the primary care setting’s potential to enhance access to evidence-based treatment, our team developed a modification of FBT, Family-Based Treatment for Primary Care (FBT-PC), for delivery in primary care by primary care providers (pediatric and family physicians, nurse practitioners, and physician’s assistants) [22]. While we preserved the main principles of FBT, we modified several aspects to improve its feasibility for delivery by primary care providers [22]. Precedent has been established for the majority of these adaptations in another modification of FBT, Parent-Focused Treatment, which has comparable efficacy to standard FBT [4].

FBT-PC maintains core principles of FBT including emphasizing weight restoration, supporting caregivers to implement meal monitoring, separating the illness from the adolescent, and reducing caregiver and adolescent guilt and blame. A study examining the feasibility of FBT-PC for 15 patients who received this adaptation found a significant increase in BMI and comparable levels of retention (86.7%) at 3 months to treatment studies of standard FBT [22].

The purpose of the current study was to establish proof of concept for FBT-PC delivered by a primary care provider for adolescents with restrictive eating disorders. We examined improvements in BMI percentile and reduction in weight suppression from baseline to end of treatment (EOT), number of patients achieving > 95% of expected body weight (EBW), number of patients no longer meeting DSM-5 criteria for an eating disorder at EOT, as well as rates of retention in both groups.

## Methods

Retrospective chart review was used to collect information from the medical records of all child and adolescent patients who received FBT-PC or FBT for a restrictive eating disorder between May 1, 2017 and August 31, 2019 at a Midwestern medical center. Included patients had a diagnosis of anorexia nervosa or other specified feeding and eating disorder (OSFED) characterized by weight loss or failure to make expected weight gains. Patients who were in the midst of treatment at the end of this timeframe or who opted out of treatment before initiating interventions were excluded. Outside of these considerations, all potential participants were included. Eating disorder diagnoses and psychiatric co-morbidities were determined based on clinical interviews as part of routine care using the Diagnostic and Statistical Manual-Fifth Edition (DSM-5) criteria [23]. Diagnoses were made by a primary care provider in consultation with a clinical psychologist with eating disorder expertise (for the FBT-PC group) or by a clinical psychologist (for the FBT group). The study was approved by the Mayo Clinic Institutional Review Board.

Coders reviewed medical records and retrieved the following data: demographic characteristics, psychiatric and medical diagnoses, and current medications and treatments. In addition, coders extracted date, BMI, and BMI percentile from all FBT or FBT-PC visits, eating disorder diagnosis, length of eating disorder from reported onset to intake, highest historical BMI percentile, and referrals made to a higher level of care during treatment. Because medical parameters including blood pressure, pulse, and temperature were generally only taken during FBT-PC visits and not during FBT visits, this data was not extracted. In addition, coders extracted scores on the Patient Health Questionnaire Modified for Teens (PHQ-9 M) [24], assessing depressive symptoms at intake. Few patients completed this measure at EOT, and so scores from this timepoint were not extracted. Treatment usage data was also extracted, including number of minutes in treatment based on what was recorded in the clinical note, number of sessions, duration of treatment, and provider-reported outcome (e.g. treatment success, drop-out, referral to higher level of care). Weights corresponding to highest reported historical BMI percentile were calculated, and weight at intake and EOT were extracted and used to calculate weight suppression.

### Participants

#### Family-Based Treatment (FBT) cohort

Fifteen consecutive adolescent patients (Mean age = 15.7; SD = 2.0, Range 12–18 years) with restrictive eating disorders received FBT from one clinical child and adolescent psychologist certified in the intervention. The sample was almost entirely Caucasian (*n* = 14, 93%) and female (*n* = 13, 86.7%). Diagnosis at intake included anorexia nervosa restricting subtype (*n* = 11, 73.3%) and anorexia nervosa binge/purge subtype (*n* = 4, 26.7%) based on DSM-5 criteria. BMI percentile at intake was 44.2 (SD = 24.1, Range 1.4–91) and mean patient-reported highest historical BMI percentile was 73.1 (SD = 22.0, Range 23–99). Duration of illness, from onset to treatment initiation, was 10.3 months (SD = 11.2, Range 2–48 months). Average rate of weight loss was 1.5 kg per month (SD = 1.1, Range .3–3.25 kg per month). Average weight suppression, defined as the difference between the weight corresponding to the highest historical BMI percentile and current weight was 10.8 kg (SD = 8.2, Range 2–32 kg), All but 1 patient (93%) were below 95% EBW at baseline.

Many patients had one or more comorbid psychiatric diagnosis including anxiety disorders (*n* = 9), depressive disorders (*n* = 9) and post-traumatic stress disorder (*n* = 2). Nearly one half of the patients (46%; *n* = 6) were on psychiatric medications, including 5 patients on an SSRI/SNRI and 1 on multiple psychiatric medications. Two patients were being seen in individual psychotherapy for other psychiatric disorders and one was enrolled in a primary care-based care coordination program for depression. In this sample, two patients had a history of a psychiatric hospitalizations or emergency department visit for acute suicidal ideation and/or behaviors prior to enrollment. See Table 1 for patient characteristics.

**Table 1:** Participant Characteristics at Intake

Participant characteristic	FBT-PC (*n* = 15)				Standard FBT (n = 15)				*p*-value
	mean	median	IQR	SD	mean	median	IQR	SD	
Age (years)	14.7	15	2	1.9	15.7	16	3	2.0	.13
Highest historical BMI percentile	83.3	87	17	12.0	73.1	80	32	22.0	.13
BMI percentile at intake	53.4	57.0	46.6	26.2	44.2	48.6	46.8	24.1	.34
Duration of illness (months)	14.5	11.0	16.0	9.3	10.3	8.0	8.0	11.2	.27
Rate of weight loss (kg/month)	1.2	.8	1.4	1.1	1.5	1.3	1.8	1.1	.44
Weight suppression (kg)	9.5	10	3.9	4.4	10.8	8	12.5	8.2	.62
PHQ-9 score	8.6	7	13	7.2	9.2	9	12	6.5	.82
**Participant characteristic**	***n***	**%**			***n***	**%**			***p*-value**
% female	12	80			13	86.7			0.62
% white	11	73			14	93			.14
Purging symptoms	0	0			4	26.7			.03
Comorbid diagnosis (%)	10	66			10	66			1.0
Psychiatric medications	6	40			6	40			1.0
Prior hospitalization	2	13			2	13			1.0

*Note*. *IQR* Interquartile range, *BMI* Body Mass Index, *PHQ-9* Patient Health Questionnaire – 9

#### Family-Based Treatment-primary care (FBT-PC) cohort

Fifteen adolescent patients (Mean age = 14.7 years; SD = 1.9, Range 13–19 years) with restrictive eating disorders received FBT-PC from one of 4 trained primary care providers (3 board-certified pediatricians, 1 family nurse practitioner). Patients in this cohort were largely female (*n* = 12, 80%) and Caucasian (*n* = 11, 73%), followed by Asian American (*n* = 3, 20%). Eating disorder diagnoses were made based on DSM-5 criteria, in consultation with a clinical psychologist with expertise in eating disorders. The majority of the patients were diagnosed with anorexia nervosa restricting subtype (*n* = 12, 80%), followed by OSFED characterized by dietary restriction and weight loss or failure to make expected weight gain (*n* = 3, 20%). At baseline, mean BMI percentile was 53.4 (SD = 26.2, Range 4–88). The majority of patients had a history of tracking above the 50th percentile for BMI with an average highest BMI percentile of 83.3 (SD = 12.0, Range 4–88). Patients reported an average length of illness of 14.5 months (SD = 9.3, Range 1.4–91 months). Average rate of weight loss was 1.2 kg per month (SD = 1.1, Range .2–4.3 kg per month). Average baseline weight suppression was 9.5 kg (SD = 4.4, Range 3.3–19.2 kg). All patients were below 95% EBW at baseline.

The majority of patients in FBT-PC had one or more comorbid psychiatric diagnosis including anxiety disorders (*n* = 8) and depressive disorders (*n* = 11). At intake, 40% of the participants (*n* = 6) were on psychiatric medications including selective serotonin reuptake inhibitors or selective norepinephrine reuptake inhibitors (SSRI/SNRI) (*n* = 5) and one patient was on multiple psychiatric medications. In addition, 6 of the adolescents were receiving adjunct individual psychological therapy for a comorbid non-eating disorder condition (40%). Two patients had a history of a psychiatric hospitalization or emergency department visit for acute suicidal ideation and/or behaviors. See Table 1 for patient characteristics.

#### Setting

Both cohorts were comprised of local patients served by two Midwestern primary care pediatrics clinics affiliated with a major academic medical center located in a midsize city. The clinics serve a largely Caucasian (76%) population of children and adolescents who are evenly distributed with respect to gender and include a mix of rural and urban patients. The FBT-PC cohort was comprised of inter-practice referrals or patients on the primary care providers’ own panels, identified as part of routine medical care. The psychologist delivering standard FBT was affiliated with these 2 clinics in the role of an integrated behavioral health provider but saw patients as part of an independent psychology practice. Patients were triaged to either FBT-PC or standard FBT based on first clinician availability.

### Interventions

#### Standard FBT

As described in the FBT manual [1], this intervention involves three phases. Phase 1 empowers parents to facilitate weight restoration, which is necessary for successful recovery from a restrictive eating disorder [25, 26]. Phase 2 begins once weight restoration is achieved and involves transitioning developmentally appropriate control over eating back to the patient. Phase 3 begins when the patient is no longer restricting their dietary intake and centers on helping the patient re-establish healthy relationships with family members, addressing issues of adolescent development and preventing relapse. Patients in the specialty care cohort received this treatment as manualized, with the exception of no family meal, which was omitted due to clinic regulations.

FBT typically involves weekly sessions for the entirety of Phase 1. Sessions can then be spaced out to bimonthly or monthly as families transition into Phases 2 and 3. All sessions are 50–90 min. No nursing support is used for these appointments and all physiological data (height and weight) is recorded and discussed by the FBT provider. Measurements are taken in light street clothes, without shoes. The specialty provider for this cohort was certified in FBT, a process that involved 2 days of classroom training and 25 h of individual case consultation with faculty from the Training Institute for Child and Adolescent Eating Disorders.

#### FBT-PC

FBT-PC was modeled after Phases 1 and 2 of FBT [1]. Primary care providers received training during two 90-min didactic sessions delivered by clinical psychologists with specialized training in FBT and eating disorders. All FBT-PC providers participated in monthly hour-long case consultation meetings with these psychologists. Providers also utilized informal, brief consultation with the two psychologists on an as-needed basis.

Several components of FBT were modified to better align with primary care practice, including no family meal, no formal sibling involvement, and shorter visits. Nurses took non-blinded weights and heights at the start of each session (patient was in light street clothes, with no shoes). These measurements were discussed in the context of the patient’s growth trajectory with the family by the primary care provider.

Adolescents in the primary care cohort were seen for an intake appointment with their parent(s)/caregiver(s) that typically lasted 45 min. At this visit, providers educated the family about the consequences of eating disorders and worked to increase caregivers’ confidence that they could help their child overcome their eating disorder. In following visits, providers gave practical advice and helped to problem-solve ways to achieve weight restoration, including methods to maximize caloric density of meals, setting limits on exercise, and encouraging caregivers to present a united front in the face of the eating disorder. Appointments were initially scheduled on a weekly basis and then decreased in frequency based on symptom improvement per providers’ clinical judgment. Follow-up appointments were typically 30 min.

Given the substantial evidence supporting the importance of early weight gain, more specifically between 1.8–2.6 kg, within the first 4 weeks of FBT [27–29], patients in both cohorts who could not attain this target were identified as needing a more intensive level of care. In these cases, patients receiving FBT-PC were referred to either standard FBT, or a residential or intensive outpatient treatment setting. Patients receiving FBT were referred to a residential or intensive outpatient setting. Any patient who transitioned from FBT-PC to standard FBT was classified as a treatment failure.

#### Outcomes

Because FBT-PC was implemented as part of standard clinical care in the primary care setting, using pre-existing clinic workflows, no eating disorder assessments were administered. For the purpose of this study, we examined weight gain through improvement in BMI percentile and reduction in weight suppression. The latter was calculated as the difference between the weight that corresponded to the patient’s highest historical BMI percentile and their current weight.

We also examined the percentage of patients who met benchmarks of clinical improvement including no longer meeting DSM-5 criteria for an eating disorder diagnosis. This included elimination of dietary restriction, reversal of low body weight, as well as resolution of cognitive symptoms including: fears of getting fat, body image disturbance, disproportionate impact of weight/shape on self-worth, and/or failure to recognize the severity of the eating disorder symptoms. For the FBT-PC condition, this determination was made by the FBT-PC provider in consultation with a clinical psychologist with expertise in eating disorders. For the FBT condition, this determination was made by the clinical psychologist providing the intervention.

Finally, we evaluated the percentage of patients who achieved weight restoration to > 95% Expected Body Weight (EBW) defined as the weight corresponding to the BMI percentile where the patient previously tracked. The BMI percentile that corresponded to 100% EBW was determined through review of the patient’s historical data on CDC growth charts by two independent raters (C.H.W. and L.S.) blind to patient treatment group. EBW estimates between raters that were within 5 BMI percentiles were considered reliable and the average of these two values was used as the value for 100% EBW. Discrepancies between these raters were resolved by consensus with disagreements resolved with input by a third party (J.L.). The intraclass correlation for the inter-rater reliability of EBW was .979 with 95% CI (0.956, 0.990).

Independent samples t-tests were used to compare participants in the FBT-PC and FBT cohorts on age, highest historical BMI percentile, baseline BMI, and duration of illness. Chi-square analyses were used to examine differences in gender, baseline eating disorder diagnoses and baseline psychiatric diagnoses. We additionally used Chi-square analyses to examine the differences in the number of patients who achieved > 95% EBW and the number of patients who no longer met DSM-5 criteria for an eating disorder.

We used independent samples t-tests to examine change in BMI percentile from pre- to post-treatment and reduction in weight suppression and calculated Cohen’s d to examine effect sizes for these continuous variables.

## Results

### Demographic characteristics

We found no significant between-group differences at baseline on any variables except for purging behaviors, with more patients in the FBT group endorsing these behaviors as part of their symptom profile, *χ*^2^ = 4.62; *p* = .03. See Table 1 for descriptive statistics.

### Outcomes

In terms of weight, each group showed large increases from baseline to EOT in BMI percentile (FBT-PC, *t* = − 5.43; *p* < .0001; standard FBT, *t* = − 6.02; *p* < .0001). In both groups, effect sizes for increased in BMI percentile exceeded Cohen’s convention for a large effect (FBT-PC: *d* = .94; standard FBT: *d* = 1.15). Weight suppression also significantly improved throughout treatment percentile (FBT-PC, *t* = 8.07; *p* < .0001; standard FBT, *t* = 6.15; *p* < .0001), with large effect sizes for reduction in weight restoration (FBT-PC: *d* = 1.83; standard FBT: *d* = 1.21) (Table 2).

**Table 2:** Effect of treatment on weight from baseline to end of treatment

	Groups									
	FBT-PC					FBT				
	n	Pre Mean (SD)	Post Mean (SD)	t-value	Cohen’s *d*	n	Pre Mean (SD)	Post Mean (SD)	*t-*value	Cohen’s *d*
BMI percentile	10	51.0 (27.4)	73.5 (20.0)	-5.43*	.94	9	48.5 (27.1)	74.5 (16.9)	-6.02*	1.15
Weight suppression (kg)	10	9.14 ( 4.3)	2.76 (2.4)	8.07*	1.83	9	11.05 ( 9.1)	1.66 (6.2)	6.16*	1.21

*P < .0001

At EOT, 10 patients in the FBT-PC cohort and 9 patients in standard FBT no longer met DSM-5 criteria for an eating disorder (*χ*^2^ = .144, OR = .75, *p* = .7). With regards to weight restoration to > 95% EBW, 12 patients in FBT-PC and 13 patients in FBT met that benchmark (*χ*^2^ = .240, OR = 1.63, *p* = .62) (Table 3).

**Table 3:** Percentage of patients meeting benchmarks of clinical improvement at end of treatment

	Cohort				*χ*^2^	OR	95% CI	*z*-value	*p*-value
	FBT-PC (n = 15)		Standard FBT (n = 15)						
	*n*	%	*n*	%					
DSM-5 criteria	10	67	9	60	0.14	0.75	0.17 to 3.32	0.38	0.71
95% Expected Body Weight	12	80	13	87	0.24	1.63	0.23 to 11.46	0.49	0.63

**Fig. 1:**
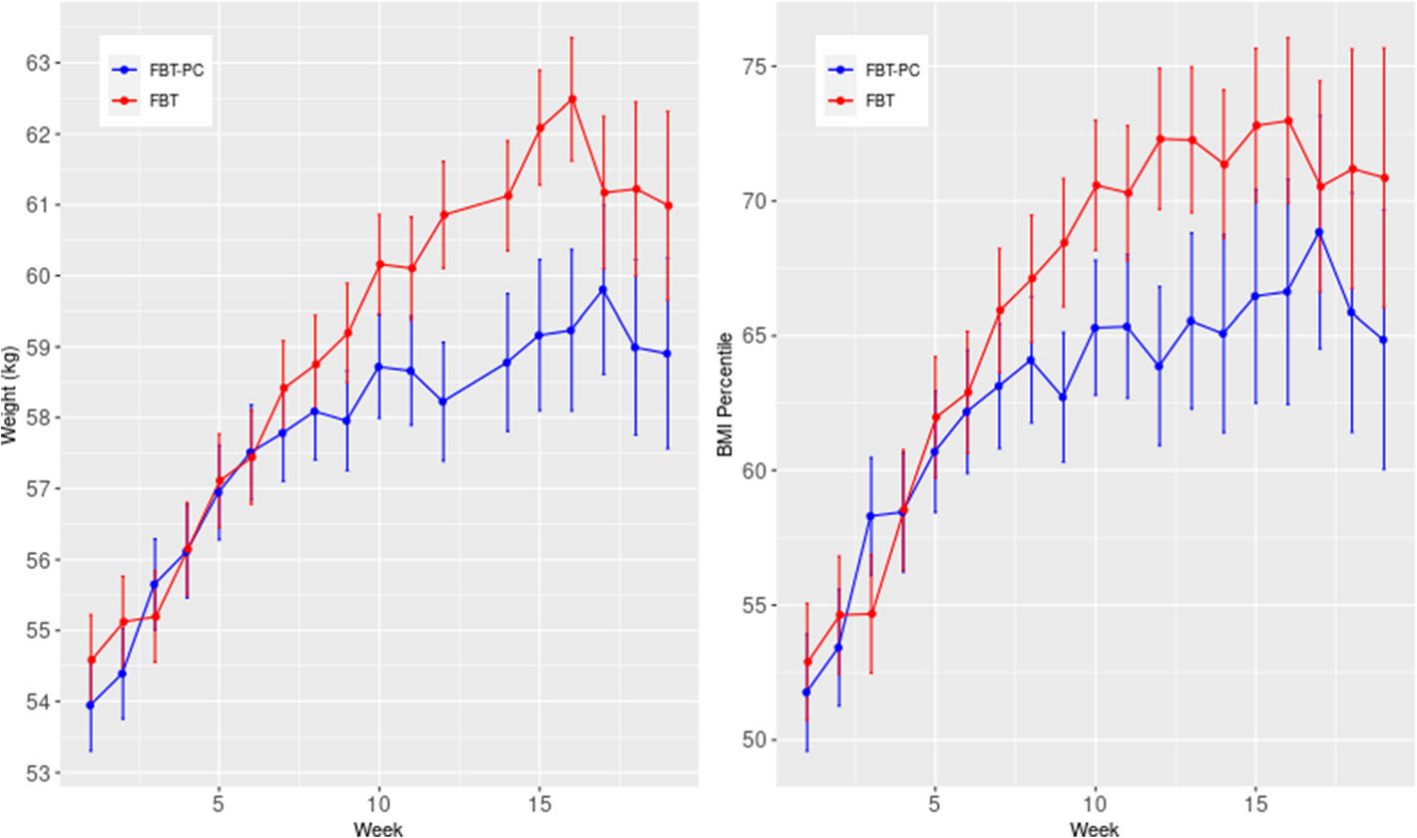
Increases in weight and BMI percentile over time for both cohorts

In the first four weeks of treatment, there were no differences between the cohorts in weight gain, with FBT-PC achieving an increase of 2.1 kg (SD = 1.5) and FBT achieving an increase of 1.6 kg (SD = 2.1), *t* [24]=.752, *p* = .55. When only considering patients who completed treatment, patients in each cohort achieved an average weight gain of 2.1 kg in the first four weeks (Please see Fig. 1).

There were no differences in the number of dropouts, with 3 patients in each cohort dropping out of treatment. The number of patients referred to a higher level of care also did not differ significantly (FBT-PC = 2; FBT = 3, *χ*^2^ = .25; *p* = .88). During treatment, 4 patients in FBT-PC were referred to a primary care-based care coordination program for depression compared to one patient in standard FBT (*χ*^2^ = 2.16, *p* = .14). In addition, one patient in FBT-PC was referred to a teen depression therapy group while no patients in standard FBT were referred for individual or group therapy (*χ*^2^ = 1.03, *p* = .31). Two patients in FBT-PC were seen by an integrated behavioral health social worker for a single crisis triage session to assess for suicidality, but neither were found to be at risk and no further services were recommended. One patient was referred to a psychological consultation. However, it was determined additional services were not needed for mental health concerns. No patients in FBT were referred for crisis or mental health assessment (*χ*^2^ = 2.1, *p* = .14). Finally, two patients in FBT-PC were prescribed medications during the course of eating disorder treatment and no patients in standard FBT were prescribed medications with no significant differences between the treatment conditions, *χ*^2^ = 2.14, *p* = .14).

Independent samples t-tests were used to examine differences in the number of sessions and time spent in sessions. In terms of treatment characteristics, the number of sessions attended (FBT-PC Mean = 12.5, standard FBT Mean = 13.6) and length of treatment in months (Both groups Mean = 6.5) were comparable, but patients in standard FBT spent significantly more time in session (Mean = 668.7 min, SD = 301) than those in FBT-PC (Mean = 366.3 min; SD = 181) *t* = 3.32, *p* =,00. See Table 4 for a summary of treatment characteristics.

**Table 4:** Differences in treatment characteristics, drop-outs, and referrals between the two cohorts

	Cohort					
	FBT-PC (n = 15)		Standard FBT (*n* = 15)			
	mean	SD	mean	SD	*t*-value	*p*-value
Weight gain in first 4 weeks	2.1	1.5	1.6	2.1	0.75	.55
Number of FBT visits/sessions	12.5	6.2	13.6	6.17	−0.50	.62
Length of treatment (months)	6.5	3.5	6.5	2.82	−.06	.96
Number of session minutes	366.3	181.1	668.7	301.75	3.32	.00
	***n***	**%**	***n***	**%**	***χ***^2^	***p*-value**
Drop outs	3	20	3	20	0	1.0
Referred to higher level of eating disorder care	2	13	3	20	.25	.88
Referred to primary care depression care coordination	4	27	1	7	2.16	.14
Referred to psychotherapy (individual or group)	1	7	0	0	1.03	.31
Referred for mental health/ crisis assessment	3	20	0	0	2.1	.14
Prescription of psychiatric medication	2	13	0	0	2.14	.14

## Discussion

Due to a lack of qualified providers, many children and adolescents with eating disorders are unable to initiate evidence-based treatment. Findings from this study provide preliminary evidence that primary care providers may have a role in supporting weight restoration and recovery for adolescents with restrictive eating disorders. Based on these findings, additional empirical evaluation of a primary care-based eating disorder intervention, FBT-PC, is warranted. In our evaluation, both cohorts had large improvement in BMI percentile and reduction in weight suppression. Using categorical benchmarks of clinical improvement, at EOT, 67% of patients in the FBT-PC cohort no longer met DSM-5 criteria for an eating disorder and 80% achieved weight restoration to at least 95% EBW, which were comparable to the rates seen in the standard FBT cohort (60 and 87%, respectively). FBT-PC had a similar retention rate to FBT, which was also comparable to that of several large randomized controlled trials of FBT [2–6]. In addition, similar numbers of patients in each treatment group were referred to a more intensive level of eating disorder care.

One-third of patients in the FBT-PC group were referred to mental health assessment and case management, with the largest group being referred to a primary care-based depression care coordination program. This care coordination program is available to all primary care providers in the practice and uses nurses, supervised by child psychiatrists, to provide case management and consultation regarding patient depression and, as needed, assistance with medication management. It is not intended to serve as a therapy replacement. Because there were no significant differences in baseline levels of depressive symptoms as measured by the PHQ-9 M, this referral pattern is unlikely to reflect a cohort difference in the level of mental health comorbidities. It is possible this reflects a difference in amount of patient distress during treatment, or, alternately, a difference in clinician training and comfort with the high level of patient distress typically seen in patients with eating disorders, particularly when undergoing weight restoration. Further study is necessary to examine whether adjunct depression care coordination is a beneficial component of FBT-PC. Although it is possible that these adjunct services play an important augmentative role when eating disorder treatments are delivered by non-mental health providers, there are also potential disadvantages of offering services targeting mood during the weight restoration phase of treatment. In particular, additional mental health treatments may dilute families’ resources for and focus on weight restoration and psychotropic medications have minimal benefit in underweight patients [30].

Patients in each group attended similar numbers of treatment sessions over a similar number of months. However, patients in FBT-PC achieved gains in weight in half the total visit time. As such, FBT-PC may represent an efficient option to effectively address some of the gaps in access to care for young patients with eating disorders and has particular potential for healthcare systems with limited resources for eating disorder treatment.

Although the retrospective design did not allow for randomization, there were no significant differences between cohorts on age, gender, presenting BMI percentile, historical highest BMI percentile, or length of illness. In both study cohorts, patients were psychiatrically complex and scored above clinical cutoffs for depression. In particular, the majority in each group had one or more comorbid mental health diagnosis and a small yet notable number had experienced a psychiatric hospitalization or emergency room visit for suicidal behavior. Diagnostically, the primary difference between the cohorts was that over a quarter of the patients in the standard FBT group reported purge behaviors, compared to none in the primary care group. Because the primary limitation of the study was the lack of standardized eating disorder measures, it is difficult to know whether patient complexity differed in a meaningful way. In spite of this, at minimum, FBT-PC may represent a viable option for patients with relatively straightforward or early illness profiles or as a stopgap measure to prevent further deterioration for patients awaiting specialty care. Further, although FBT-PC was not designed to be a replacement for FBT, but rather as a lower-step option for care, the findings that both cohorts were similar in terms of amount of weight loss and presenting BMI suggests that this primary care-based intervention may also be a potential alternative in circumstances where outpatient eating disorder treatment is not available.

Though patients spent less time in session in FBT-PC than in standard FBT, the former intervention is still time-intensive for a primary care practice, where time and resources are scarce, and providers are over-paneled. Despite the effort required, many of these patients will present regularly to primary care regardless, for management of medical complications secondary to their disorders, such as amenorrhea, abdominal pain, fatigue, or dizziness as well as for management of general psychiatric concerns [31]. As such, combining management of these symptoms with interventions to assist patients with weight restoration may mitigate downstream effects, including burden on the healthcare system.

The primary limitation of this study is the lack of structured eating disorder measures. We are therefore limited in our ability to draw conclusions about the clinical characteristics of the treated population. A related limitation was the lack of an independent rater to assess DSM-5 criteria. Additional limitations of this study include the small sample size and retrospective cohort design. Given that the differences in the cohorts were outcomes of interest, matching samples was not reasonable. Because the standard FBT cohort included more patients with purging behaviors it is possible that this cohort may have had different or more serious eating disorder pathology. Unfortunately, the small sample size precluded any analyses of differences between those patients with purging and those without. Similarly, given the higher BMI percentiles in study patients, it is unclear how these findings generalize to lower BMI cohorts. Finally, due to the small sample size and lack of mental health data, we were not able to examine factors related to referrals to adjunct mental health services, as well as the influence these referrals may have had on treatment outcomes.

## Conclusions

The findings of this study suggest that the FBT-PC proof-of-concept is valid. Additional testing of the model on a larger scale to examine the feasibility and comparative effectiveness of FBT-PC is indicated. Although further evaluation is necessary to establish the efficacy of FBT-PC, these findings demonstrate that primary care providers have potential to help young patients restore weight and improve clinical status. This integrated approach may allow us to reach patients and families earlier in the disease progression to improve outcomes and decrease burden on patients, families and the healthcare system.

## Data Availability

The datasets analyzed during the current study are available from the corresponding author on reasonable request.

## Abbreviations

BMI: Body Mass Index
EBW: Expected Body Weight
EOT: End of treatment
FBT: Family-Based Treatment
FBT-PC: Family-Based Treatment for Primary Care
OSFED: Other specified feeding and eating disorder
SNRI: Selective norepinephrine reuptake inhibitor
SSRI: Selective serotonin reuptake inhibitor
